# Risk Stratification of Thyroid Nodules 10 mm in Diameter or Less: Strength and Pitfalls of the Ultrasonographic Assessment From a Cross-Sectional Study

**DOI:** 10.1155/ije/4063672

**Published:** 2025-09-19

**Authors:** Giuseppe Lisco, Anna De Tullio, Vito Angelo Giagulli, Giuseppina Renzulli, Vincenzo Triggiani

**Affiliations:** ^1^Interdisciplinary Department of Medicine–School of Medicine, University of Bari Aldo Moro, Piazza Giulio Cesare 11, Bari 70124, Italy; ^2^Department of Precision and Regenerative Medicine and Ionian Area (DiMePre-J)–Unit of Pathology, University Medical School of Bari, Piazza Giulio Cesare 11, Bari 70124, Italy

## Abstract

**Background:** The selection of thyroid nodules ≤ 10 mm requiring characterization and treatment should be improved, as extensive detection, cytological assessment, and surgery of small and well-differentiated thyroid carcinoma are not cost-effective.

**Aim:** To assess the accuracy of algorithms and ultrasonographic characteristics in selecting actual high-risk thyroid nodules ≤ 10 mm.

**Methods:** A cross-sectional study was conducted on 38 of 112 outpatients who attended the University of Bari and underwent echo-assisted FNA for cytological characterization of thyroid nodules ≤ 10 mm (65 out of 118) and thyroid surgery from January 01 to December 31, 2016.

**Results:** The median age of patients was 49.5 years [16; 69]. Thyroid cytology (SIAPeC-IAP 2014) was classified as TIR1 (one nodule), TIR2 (15), TIR3A (7), TIR3B (10), TIR4 (8), and TIR5 (24). Thirty-nine thyroid nodules were diagnosed as well-differentiated thyroid microcarcinoma. The clinical performance of 4 algorithms widely employed in clinical practice was low (AACE/ACE/AME, 38%; ACR-TIRADS, 45%; K-TIRADS, 60%; EU-TIRADS, 66%). Ultrasonographic features indicating high-risk nodules were hypoechogenicity (*p*=0.0047), irregular margins (*p*=0.004), and microcalcifications (*p*=0.0019). Multivariable analyses indicated that hypoechogenicity was the main ultrasonographic characteristic associated with high-risk nodules (OR = 5.48, *p*=0.0484).

**Discussion:** Validated algorithms fail to select thyroid nodules ≤ 10 mm for which cytological characterization is needed. Our results are expected to improve the reliability of current algorithms by improving the weight of variables associated with a more consistent risk of thyroid malignancy in nodules ≤ 10 mm.

## 1. Background

The prevalence of thyroid nodules has increased over the last 4 decades, leading clinicians to resort to cytological characterization more frequently, with a remarkable increase in the number of patients undergoing thyroid fine needle aspiration (FNA) and surgery because of indeterminate, suspicious, or malignant thyroid cytology [1]. However, malignant cytology represents a minority of findings (5%) among diagnostic FNA [2]. Moreover, the mortality rate of thyroid cancer is very low, indicating that intensifying thyroid nodule detection, characterization, and intervention is not cost-effective [3]. The reason for this phenomenon can be attributable to the fact that a substantial increase in the number of thyroid nodules equal to or less than 10 mm (mostly less than 5 mm) incidentally diagnosed has been observed because of the widespread use of high-frequency ultrasound over the last decades [4]. Epidemiological data show that most of these nodules are frequently indolent, even in the case of differentiated thyroid carcinoma, indicating that well-differentiated microcarcinoma usually has a favorable prognosis.

On this basis, most guidelines recommend that nodules ≤ 10 mm should be excluded from FNA and cytological assessment to reduce the rate of overdiagnosis of small (and possibly indolent) thyroid carcinomas [5]. At the same time, accumulating evidence indicates that the surveillance of well-differentiated thyroid microcarcinoma, after cytological diagnoses, can be an alternative strategy to thyroid surgery [5]. However, a small but not negligible proportion of thyroid nodules are known to progress over time in terms of size enlargement of nodules or lymph node metastatic spread, requiring prompt thyroidectomy/lymphadenectomy and radioiodine [6]. It is the case of tall-cell, columnar-cell, and hobnail-cell carcinomas that have a more aggressive clinical behavior regardless of the nodule size [7]. So, there is a real need to identify “high-risk” micronodules requiring cytological characterization and surgical management.

Several scores and algorithms have been validated to guide clinicians in selecting thyroid nodules requiring cytological characterization based on clinical and ultrasonographic characteristics [8–13]. These algorithms work by incorporating ultrasonographic features of thyroid nodules associated with a high risk of malignancy, including composition, margins, shape, echoic foci, and vascular signals [14, 15]. The appropriate use of algorithms requires specific expertise in terms of adequate use of standardized reporting systems of neck ultrasound, education of users on risk stratification systems and updates, and reminders of use at the point of care [16]. As another issue, the performance of algorithms is different, and clinicians should be aware and updated before making appropriate decisions [17–19]. Last, recommendations on the management of thyroid nodules ≤ 10 mm are less clear, with most guidelines suggesting a wait-and-see approach with no indication to FNA unless high suspicion exists. Consequently, most of these algorithms have not been designed to assess the risk of thyroid nodules ≤ 10 mm, making the decision to either perform or not perform an FNA a challenge [20].

This is a cross-sectional study aiming to address the overall performance of validated algorithms and the weight of ultrasonographic characteristics of thyroid nodules in properly conducting the clinical decision to carry out or not thyroid FNA and cytology in unselected nodules ≤ 10 mm.

## 2. Methods

### 2.1. Study Design

A cross-sectional study was conducted on 112 unselected consecutive outpatients who attended the Azienda Ospedaliera Policlinico—University of Bari from January 01 to December 31, 2016, and underwent echo-assisted FNA for cytological characterization of one or more thyroid nodules. All of them underwent thyroid surgery too. Detailed information about the study's characteristics, population, and methods is described elsewhere [21].

### 2.2. Data Extraction

We extracted data of patients with thyroid nodules ≤ 10 mm from 118 thyroid nodules of 112 outpatients examined in 2016. Overall, 65 thyroid nodules from 38 outpatients were included (nodules/outpatient ratio = 1.7).

### 2.3. Neck Ultrasound

All patients underwent neck ultrasound before FNA to collect proper information on the thyroid gland, number, site, and ultrasonographic characteristics of thyroid nodules and lymph nodes. Neck ultrasound was carried out by a single operator (VT) with ESAOTE MyLab25, 13-4 MHz linear array transducer.

### 2.4. Reporting System of Thyroid Ultrasound

Ultrasound images collected from each patient included (a) full thyroid images (right lobe, left lobe, and isthmus), (b) measurements of the three major thyroid axes in both planes (transverse and longitudinal), and (c) nodule images describing the three major nodular axes in both planes (transverse and longitudinal).

A detailed description of thyroid nodule characteristics was reported according to a standardized reporting system, including (a) nodule diameter, (b) composition, (c) echogenicity, (d) shape, (e) margins, (f) echoic foci, (g) peri-nodular calcification, and (h) vascular signals. The position of thyroid nodules was also described (lobes or isthmus; upper, inner, or lower side; anterior or posterior location; location with respect to thyroid capsule).

### 2.5. Risk Classification of Thyroid Nodules

Neck images were retrospectively re-examined by two operators (Giuseppe Lisco and Vincenzo Triggiani) for a second look. Each operator reviewed neck ultrasound images to collect information properly and run algorithms. Thyroid nodule descriptions were according to the revised ultrasound Lexicon for thyroid nodule [22].

Four algorithms were selected to estimate the risk of each nodule, namely the American Association of Clinical Endocrinology/American College of Endocrinology/Associazione Medici Endocrinologi (AACE/ACE/AME), the American College of Radiology Thyroid Imaging Reporting and Data System (ACR-TIRADS), the European TIRADS (EU-TIRADS), and the Korean TIRADS (K-TIRADS).

### 2.6. Thyroid Cytology

Upon FNA was performed, specimens were used to prepare 96% ethyl alcohol thin-layer slides for cytological assessment; then, cytological assessment was carried out by a single operator GR at the Pathology Department of the University of Bari. Most importantly, the pathologist received a detailed description of each nodule.

Cytological diagnoses were formulated according to the joint classification of the Società Italiana di Anatomia Patologica e Citologia (SIAPeC)—International Academy of Pathology (IAP) 2014 for the classification and reporting of thyroid cytology [23].

### 2.7. Thyroid Surgery

Patients with indeterminate, suspicious, or malignant lesions at cytology were referred to a surgeon. Seventeen nodules (26%) from 11 outpatients had no cytological indication of thyroid surgery. However, these nodules were part of multinodular thyroid disease or goiter, in which at least one nodule had an indeterminate, suspicious, or malignant cytology, or there was clinical indication for thyroidectomy (e.g., compressive complaints).

### 2.8. Thyroid Pathology

Thyroid specimens were sent to the Pathology Department, where the same operator (GR) handled them for pathological analyses. Most importantly, each nodule was identified and analyzed for an accurate pathological diagnosis.

### 2.9. Statistical Analysis

Descriptive statistics of quantitative variables were presented as mean ± standard deviation or median [min; max] according to their distribution. Descriptive statistics of qualitative categorical variables were presented as counts and percentages.

Comparisons between categorical variables, differences in frequencies, and odds ratios between categorical variables were assessed by the chi-square test or Fisher's exact test.

Generalized linear models were used to provide regression analysis and analysis of variance for multiple independent variables by one dependent variable (histological diagnosis) with the aim of testing for predicting ultrasonographic features identifying high-risk thyroid nodules.

All analyses were carried out with R. The statistical significance level was set to a *p* value < 0.05.

### 2.10. Sample Size

The sample size was calculated for the primary outcome which was the estimation of predicting factors (and related odds ratios) of risk of thyroid malignancy based on the leading neck ultrasonographic features (seven variables). The sample size should be intended as the sufficient number of thyroid nodules, and not patients, to include for the analyses. It was estimated with G∗power 3.1, resulting in 61 nodules (*z*-test, logistic regression, one tail, OR 6, α-error 0.05, 1 − *β* error 0.95, binomial distribution). Therefore, the sample size is adequate for the study purpose.

## 3. Results

### 3.1. Characteristics of the Study Population

The median age of the study population was 49.5 years [16; 69], 33 women (86.8%) and 5 men (13.2%).

Eighteen patients (47.3%) had a family history of nonmalignant thyroid diseases, such as chronic autoimmune thyroiditis, diffuse or multinodular goiter, and thyroid nodule(s). A family history of differentiated thyroid cancer was registered in 8 of 38 outpatients (21%).

Four patients had multinodular thyroid disease with or without thyroid enlargement (10.5%); the remaining 34 (89.5%) had a single thyroid nodule. Ten of 38 patients (26.3%) had at least one palpable nodule, and half (5 outpatients) had hard palpable nodules. None had lateral cervical lymph node enlargement at physical examination, and the ultrasonographic assessment did not reveal images of suspicious lymph nodes.

A basic thyroid panel test was available in 34 out of 38 patients. Fifteen (39.5%) were on levothyroxine. The median TSH value was 1.65 mIU/L [0.6; 18]. Among them, two patients had a TSH value suggestive of hypothyroidism and were not well controlled while on levothyroxine replacement. None had hyperthyroidism at the time of FNA, while 10 individuals (26.3%) had concomitant chronic autoimmune thyroiditis.

Nine patients (23.7%) had a recent measurement of unstimulated serum calcitonin, and all values were within the normal reference range.

### 3.2. Ultrasonographic Features of Examined Nodules

#### 3.2.1. Diameter

Thyroid nodule diameters ranged from 4 to 10 mm (median value of 8 mm). Among them, 6 (9.2%) had a diameter ≤ 5 mm (4 of 5 mm and 2 of 4 mm), while the remaining 59 (90.7%) had a diameter > 5 mm.

#### 3.2.2. Composition

Sixty-three of 65 (96.7%) nodules had a solid composition, while the remaining 2 (3.3%) had a mixed solid-cystic composition with a mural eccentric solid component.

#### 3.2.3. Echogenicity

Echogenicity was described as follows: 4 nodules (6.1%) were hyperechoic, 7 (10.8%) isoechoic, 24 (36.9%) hypoechoic or slightly hypoechoic, and 30 (46.2%) markedly hypoechoic.

#### 3.2.4. Shape

Fifty-two of 65 nodules had an oval or round shape (80%), while the remaining 20% (13 out of 65) had a “taller than wide” shape.

#### 3.2.5. Margins

Around half of the nodules (33, 50.7%) had regular margins. Six (9.2%) had irregular round-shaped margins, and 18 (27.7%) had irregular spiculated margins. Last, 8 nodules (12.1%) had ill-defined margins.

#### 3.2.6. Echoic Foci

Thirty-seven (59.6%) nodules did not have echoic foci. Nineteen (29.2%) had echoic foci attributable to microcalcifications. Seven (10.8%) were classified as having difficult-to-characterize echoic foci, while the remaining 2 (3%) had rough inner macrocalcifications.

#### 3.2.7. Peri-Nodular Calcifications

Only one nodule of 8 mm in diameter has an interrupted peri-nodular calcification.

#### 3.2.8. Echo-Color-Doppler

Vascular signals were evaluated with Echo-Color/Power Doppler. Most nodules had no vascularization or exhibited slight continuous or discontinuous peri-nodular signals (54, 83.1%). The remaining 11 (26.9%) had peri- and intranodular vascular signals.

### 3.3. Risk Classification of Thyroid Nodules

Thyroid nodules were classified according to 4 systems: two from the US, one European, and one Korean.

According to the AACE/ACE/AME, thyroid nodules were classified into three classes of risk (low, indeterminate, or high risk). More precisely, 7 had low risk of malignancy (US1), 16 had indeterminate risk (US2), and the remaining 43 were at high risk of malignancy (US3).

According to the ACR-TIRADS, thyroid nodules were classified as follows: one was TR2 (not suspicious), 8 were TR3 (mildly suspicious), 28 were TR4 (moderately suspicious), and 28 were TR5 (highly suspicious).

According to the EU-TIRADS, thyroid nodules were classified as follows: one was EU-TR2 (risk of malignancy = 0, benign), 8 were EU-TR3 (risk of malignancy 2%–4%, low risk), 13 were EU-TR4 (risk of malignancy 6%–17%, intermediate risk), and 43 were EU-TR5 (risk of malignancy 26%–87%, high risk).

Last, according to the K-TIRADS, nodules was classified as follows: none was K-TR2 (benign), 8 were K-TR3 (low suspicious), 28 were K-TR4 (moderate suspicious), and 29 were K-TR5 (high suspicious).

### 3.4. Cytological Findings

Thyroid cytology was classified and reported as follows: one nodule was classified as TIR1 (nondiagnostic cytologic), 15 were TIR2 (benign cytology), 17 had indeterminate cytology (7, low-risk indeterminate lesions, or TIR3A; 10, high-risk indeterminate lesions, or TIR3B), 8 were classified as suspicious of malignancy (TIR4), and 24 were cytologically defined as malignant lesions (TIR5).

### 3.5. Pathological Findings

Thirty-nine (60%) thyroid nodules were definitively diagnosed as differentiated thyroid microcarcinoma (33 papillary and 3 follicular thyroid cancer), while the remaining 26 (40%) were definitively diagnosed as nonmalignant nodules (goiter, adenoma, goiter and thyroiditis, and thyroiditis). Most importantly, none of nodules diagnosed as differentiated carcinoma had a suspicious histological subtype; so these were defined as well-differentiated thyroid microcarcinoma [7].

### 3.6. Performance of Thyroid Cytology

The performance of thyroid cytology was estimated since around 80% of patients underwent thyroid surgery because of cytological judgment. Thyroid cytology showed high sensitivity (95%) and moderate specificity (80%), with an overall clinical accuracy of 88% (Table 1).

### 3.7. Retrospective Analyses of the Performance of Algorithmic-Based Decisions to Carry Out or Not Thyroid FNA

#### 3.7.1. Selection of Thyroid Nodules According to the AACE/ACE/AME Algorithm

We retrospectively calculated which nodules would have been recommended for cytological characterization in a hypothetical scenario in which the algorithms would have made any decision.

According to the AACE/ACE/AME algorithm, 41 of 65 nodules (63.1%) would have been suitable for cytological characterization (class US3), while 24 (36.9%) would not have been suitable for FNA (classes US1 and US2).

#### 3.7.2. Selection of Thyroid Nodules According to the ACR-TIRADS Algorithm

According to the ACR-TIRADS algorithm, 38 of 65 nodules (58.5%) would not have been suitable for cytological characterization (classes TR2, TR3, TR4, and one TR5 < 5 mm), while 27 (41.5%) would have been suitable for FNA (all the other TR5).

#### 3.7.3. Selection of Thyroid Nodules According to the EU-TIRADS Algorithm

According to the EU-TIRADS algorithm, 23 of 65 nodules (35.4%) would not have been suitable for cytological characterization (classes TR2, TR3, TR4, and one TR5 < 5 mm), while 42 (64.6%) would have been suitable for FNA (all the other TR5). It should be considered that the algorithm advises that nodules with high suspicious risk (TR5) ranging from 5 to 10 mm can be either followed up or undergo FNA.

#### 3.7.4. Selection of Thyroid Nodules According to the K-TIRADS Algorithm

According to the K-TIRADS algorithm, 28 of 65 nodules (43.1%) would not have been suitable for cytological characterization (classes TR3, TR4, and one TR5 < 5 mm), while 37 (56.9%) would have been suitable for FNA (all the other TR5).

### 3.8. Level of Concordance Amongst the 4 Algorithms

The 4 algorithms would have recommended not to perform thyroid FNA concordantly only in 3 nodules (A, B, C), which were histologically diagnosed as thyroid papillary carcinoma (Supporting Information, Table S1).

The 4 algorithms would have recommended to perform thyroid FNA concordantly in 9 nodules; six (D, E, F, G, H, I) were histologically diagnosed as thyroid papillary carcinoma (Table S2).

### 3.9. Performance of the 4 Algorithms (AACE/ACE/AME, ACR-TIRADS, EU-TIRADS, K-TIRADS)

The performance of the 4 algorithms was calculated by considering thyroid pathology as the gold standard.

The AACE/ACE/AME algorithm showed very low sensitivity and specificity, with an overall clinical accuracy of 38% (Table 2).

The ACR-TIRADS algorithm showed very low sensitivity and specificity, with an overall clinical accuracy of 45% (Table 3).

The EU-TIRADS algorithm showed low sensitivity and specificity, with an overall clinical accuracy of 66% (Table 4).

The K-TIRADS algorithm showed low sensitivity and specificity, with an overall clinical accuracy of 60% (Table 5).

### 3.10. Ultrasonographic Features of Thyroid Nodule Associated to High Risk of Malignancy

Upon the performance of the 4 algorithms has been analyzed, a specific estimation of the risk of thyroid malignancy for each ultrasonographic nodule feature was calculated.

Solid compared to the mixed composition was found to be related to high-risk thyroid nodules, even if the result was not statistically significant (Table S3; Fisher's Exact Test for Count Data with alternative hypothesis = true odds ratio is not equal to 1: *p* value = 0.1562; OR = +∞; 95% IC [0.28; +∞]).

Hypoechoic (both slightly and markedly hypoechoic) nodules were found to be associated with a high risk of thyroid malignancy compared to hyperechoic or isoechoic nodules (Table 6, OR = 9.4; 95% CI [1.69; 98.97], *p*=0.0047).

Nodules with a “taller than wide” shape, compared to those with round or ovoidal shape, had a similar risk of thyroid malignancy (Table S4; Fisher's Exact Test for Count Data with alternative hypothesis = true odds ratio is not equal to 1: *p* value = 1; OR = 1.1; 95% CI [0.27; 4.82]).

Nodules with irregular than regular or ill-defined margins had a significantly higher risk of thyroid malignancy (Table 7, OR = 5.6; 95% CI [1.52; 26.7], *p*=0.004).

Nodules with microcalcification compared to those without or with any of less specific echoic foci had a markedly higher risk of thyroid malignancy (Table 8, OR = 8.9; 95% IC [1.80; 89.19], *p*=0.0019).

Nodules with peri- and intranodular vascular flow compared to those with absent or peripheral vascularization had a slightly higher risk of thyroid malignancy, but the result was not statistically significant (Table S5; Fisher's Exact Test for Count Data with alternative hypothesis = true odds ratio is not equal to 1: *p* value = 0.503; OR = 1.96; 95% IC [0.4; 12.7]).

### 3.11. Ultrasonographic Features Predictive of Thyroid Nodule Malignancy

A logistic regression model was utilized to identify the predicting factors associated with thyroid nodule malignancy.

Two specific models were utilized to analyze the phenomenon. In model 1, all ultrasonographic features were included. As shown in Table 9, model 1 did not identify any specific feature as a predicting factor of thyroid malignancy.

In model 2, only ultrasonographic features associated with a higher risk of thyroid malignancy were included (3 covariates). As shown in Table 10, the model described a statistically significant association between the risk of thyroid malignancy and high-risk ultrasonographic features, and the leading contributors to this association were hypoechoic or markedly hypoechoic nodules (OR = *e*^*β*1^ = *e*^1.7021^ = 5.48, *p*=0.0484).

## 4. Discussion

An adequate selection of thyroid nodules ≤ 10 mm requiring FNA and cytological characterization is essential to avoid unnecessary and wasteful procedures, decrease the risk of overdiagnosis, and preserve patients' quality of life. The results of our cross-sectional study suggest that algorithm-based selection of thyroid micronodules requiring cytological characterization as highly suspicious for malignancy appears insufficient compared to a less selective strategy. Our data confirmed the uncertain performance of the 4 algorithms (overall clinical accuracy: AACE/ACE/AME, 38%; ACR-TIRADS, 45%; K-TIRADS, 60%; EU-TIRADS, 66%) used in worldwide clinical practice when these are requested to run data from micronodules compared to nodules > 10 mm [24–26].

As another practical issue, the systematic use of all algorithms to calculate simultaneously the risk of each nodule appears unrealistic and time-consuming. Moreover, the 4 algorithms expressed the same judgment (100% agreement) in 9 of 65 nodules (performing FNA in 6; not FNA in 3; all were papillary carcinomas).

Ultrasonographic features indicating high-risk nodules were hypo-echogenicity (OR 9.4, *p* = 0.0047), irregular margins (OR 5.6, *p* = 0.004), and microcalcifications (OR 8.9, *p* = 0.0019). Nodules with solid composition compared to mixed composition were at higher risk of malignancy too, even if the difference was not statistically significant because of a low number of nodules with mixed composition (two). The findings are almost in line with other results [27], except for a taller-than-wide shape that, in our group of patients, did not reach a statistically relevant association as a high-risk nodule feature (OR 1.1; 95% CI [0.27; 4.82]) [14, 28]. Nevertheless, sensitive analyses assessing the role of each ultrasonographic characteristic (nodule composition, echogenicity, shape, margins, echoic foci, peri-nodular calcification, and vascular signals) on the risk of thyroid malignancy indicated that the leading factor associated with a high-risk nodule was hypoechogenicity (corrected OR = 5.48, *p* = 0.0484).

The results of our study provided with useful information to improve the selection of thyroid nodules ≤ 10 mm requiring cytological characterization [29, 30]. It is crucial to identify with ultrasound only high-risk nodules to avoid possible misdiagnoses due to the systematic implementation of current guidelines, systematically excluding most of nodules ≤ 10 mm from FNA and cytological characterization. Available algorithmic tools currently designed for that specific purpose are less accurate when dealing with thyroid nodules ≤ 10 mm than nodules >10 mm and should be improved to ameliorate the management of this cluster of patients. Our findings could be incorporated into current algorithms to adjust the weight of variables associated with a more consistent risk of thyroid malignancy before reconsidering their clinical accuracy in managing high-risk nodules ≤ 10 mm.

## 5. Study Strengths and Limitations

The strength is in the study design, conducted in the same center by experts while evaluating a group of unselected outpatients who consecutively underwent thyroid cytology and thyroid surgery. Most importantly, all nodules were aspirated by an expert endocrinologist and characterized by an expert pathologist, who consequently provided us with precise and accurate histological diagnoses. Two experts also reviewed ultrasound images to characterize the ultrasonographic features of all nodules. The choice of including patients attended at the Center in 2016, before the validation and dissemination of updated guidelines and algorithmic tools, led us to compare a more selective approach of thyroid nodule requiring cytological characterization, typically observed after 2016, with a less selective approach based on clinical expertise, typically observed before 2016.

Most of these nodules, despite being ≤ 10 mm, were categorized as at moderate-to-high suspicion according to ultrasonographic characteristics. In addition, patients had a family and personal history of risk factors for thyroid carcinoma (i.e., Hashimoto's thyroiditis and cases of differentiated thyroid cancer in relatives) with a frequency exceeding that observed in the general population but substantially in line with other reports on patients who underwent thyroid FNA [31, 32]. These data are not surprising, as the subset of our study population is typical to that observed in such a population of individuals referred to specialized centers for cytological characterization of suspicious nodules due to anamnestic, clinical, and ultrasonographic features.

The limitation is in the study nature (cross-sectional, real-life study) and related selection biases (small high-risk nodules) and in the relatively low number of nodules ≤ 10 mm as compared to the entire cohort of thyroid nodules selected with the same method in 2016 (65 out of 188), indicating that thyroid nodules ≤ 10 mm, despite being diagnosed more frequently than those > 10 mm, were less prone to be referred for FNA, also regardless to background ultrasonographic risk.

Moreover, ultrasound characteristics of nodules were retrospectively collected from frozen images, as we did not have ultrasound time frames or short video clips to review all thyroid nodules' characteristics at a glance. Last, the lack of follow-up did not allow us to register clinical outcomes and the ongoing risk stratification to compare the risk-to-benefit ratio of the two procedures (algorithm-based versus clinical-based decision).

## 6. Conclusion

Although most valuable and validated algorithmic tools fail to perform accurately in the selection of thyroid nodules ≤ 10 mm requiring cytological characterization as high-risk lesions, here, we defined the ultrasonographic hallmark of high-risk thyroid micronodules actually requiring to be referred to specialized centers for FNA and cytological characterization. The ultrasonographic feature is represented by hypoechoic/markedly hypoechoic micronodules also irrespective to other ultrasonographic features.

Besides patients with clinical characteristics suggesting thyroid FNA, those with hypoechoic and markedly hypoechoic nodules should undergo cytological characterization, even in the case of thyroid nodules ≤ 10 mm.

## Figures and Tables

**Table 1:** Performance of thyroid cytology as compared to thyroid pathology (gold standard) in driving clinical judgment to recommend or not thyroid surgery.

SIAPeC-IAP classification	Pathology	
	Malignant	Nonmalignant
Recommendation for surgery (TIR 3B, 4, or 5)	37	5
No recommendation for surgery (TIR 2 or 3A)	2	20

*Note:* Sensitivity 95%; specificity 80%; positive predictive value 88%; negative predictive value 91%; clinical accuracy 88%.

**Table 2:** Performance of the AACE/ACE/AME algorithm (thyroid pathology as the gold standard).

Algorithm-based judgment	Malignant nodules	Nonmalignant nodules
Perform FNA	20	21
Avoid FNA	19	5

*Note:* Sensitivity 51%; specificity 19%; positive predictive value 49%; negative predictive value 21%; clinical accuracy 38%.

**Table 3:** Performance of the ACR-TIRADS algorithm (thyroid pathology as the gold standard).

Algorithm-based judgment	Malignant nodules	Nonmalignant nodules
Perform FNA	15	12
Avoid FNA	24	14

*Note:* Sensitivity 38%; specificity 54%; positive predictive value 56%; negative predictive value 37%; clinical accuracy 45%.

**Table 4:** Performance of the EU-TIRADS algorithm (thyroid pathology as the gold standard).

Algorithm-based judgment	Malignant nodules	Nonmalignant nodules
Perform FNA	29	12
Avoid FNA	10	14

*Note:* Sensitivity 74%; specificity 54%; positive predictive value 71%; negative predictive value 58%; clinical accuracy 66%.

**Table 5:** Performance of the K-TIRADS algorithm (thyroid pathology as the gold standard).

Algorithm-based judgment	Malignant nodules	Nonmalignant nodules
Perform FNA	26	11
Avoid FNA	15	13

*Note:* Sensitivity 63%; specificity 54%; positive predictive value 70%; negative predictive value 46%; clinical accuracy 60%.

**Table 6:** Estimation of the risk of thyroid malignancy according to nodule echogenicity (slightly or markedly hypoechoic versus hyper- or isoechoic).

Pathology	Hyper- or isoechoic	Slightly or markedly hypoechoic	OR	*p* value
Nonmalignant	9	17	9.4; 95% CI [1.69; 98.97]	0.0047
Malignant	2	37		

**Table 7:** Estimation of the risk of thyroid malignancy according to nodule margins (irregular versus regular or ill-defined).

Pathology	Regular or ill-defined margins	Irregular margins	OR	*p*-value
Nonmalignant	22	4	5.6; 95% IC [1.52; 26.7]	0.004
Malignant	19	20		

**Table 8:** Estimation of the risk of thyroid malignancy according to echoic foci (microcalcification versus any of less specific echoic foci).

Pathology	Any of the less specific echoic foci	Microcalcifications	OR	*p* value
Nonmalignant	24	2	8.9; 95% IC [1.80; 89.19]	0.0019
Malignant	22	17		

**Table 9:** Multivariable logistic regression to predict the risk of thyroid malignancy starting from ultrasonographic features (composition, echogenicity, shape, margins, peripheral calcification, echoic foci, and vascular signals).

Ultrasonographic features	Estimate	Std. error	*z*-value	Pr (> |*z*|)
Composition	16.86	2562.21	0.007	0.9947
Echogenicity	1.6456	0.9148	1.799	0.0720
Shape	−0.8889	0.9364	−0.949	0.3424
Margins	1.5439	0.9378	1.646	0.0997
Peripheral calcifications	17.5795	3956.1804	0.004	0.9965
Echoic foci	1.0895	0.9381	1.161	0.2455
Flow	0.9327	0.8341	1.118	0.2635

*Note:* Significant codes: 0 ‘^∗∗∗^' 0.001 ‘^∗∗^' 0.01 ‘^∗^' 0.05 ‘.'.

**Table 10:** Multivariable logistic regression to predict the risk of thyroid malignancy by considering high-risk ultrasonographic features (echogenicity, margins, and echoic foci).

Ultrasonographic features	Estimate	Std. error	*z*-value	Pr (> |*z*|)
Intercept	1.6138	0.7950	−2.030	0.0424^∗^
Echogenicity	1.7021	0.8622	1.974	0.0484^∗^
Margins	0.9475	0.7353	1.289	0.1975
Echoic foci	1.3676	0.8983	1.522	0.1279

*Note:* Significant codes: 0 ‘^∗∗∗^' 0.001 ‘^∗∗^' 0.01 ‘^∗^' 0.05 ‘.'.

## Data Availability

The data supporting the findings of this study are available from the corresponding author on reasonable request.

## Nomenclature

AACE/ACE/AME: American Association of Clinical Endocrinology/American College of Endocrinology/Associazione Medici Endocrinologi
ACR-TIRADS: American College of Radiology Thyroid Imaging Reporting and Data System
EU-TIRADS: European Thyroid Imaging Reporting and Data System
FNA: Fine needle aspiration
K-TIRADS: Korean Thyroid Imaging Reporting and Data System
SIAPeC-IAP: Società Italiana di Anatomia Patologica e Citologia—International Academy of Pathology
TIRADS: Thyroid imaging reporting and data system

## Supporting Information

Tables S1–S5 and the STROBE checklist for cross-sectional studies.
