# Supplementary material for: Risk Stratification of Thyroid Nodules 10 mm in Diameter or Less: Strength and Pitfalls of the Ultrasonographic Assessment From a Cross-Sectional Study
Source: Int J Endocrinol. 2025 Sep 19;2025:4063672. doi: 10.1155/ije/4063672 (PMC12474009; doi:10.1155/ije/4063672)
Supplement: Supporting Information — Additional supporting information can be found online in the Supporting Information section. [file 4063672.f1.zip › Supplementary_tables_S1_S5.docx]

**Table S1. Characteristics of nodules for whom all the 4 algorithms would have recommended not to perform thyroid FNA**

| **Nodule** | **AACE/ACE/AME US** | **ACR-TIRADS** | **EU-TIRADS** | **K-TIRADS** | **SIAPeC-IAP 2014** | **Pathology** |
| --- | --- | --- | --- | --- | --- | --- |
| A | 1 | 2 | 4 | 4 | 3B | Papillary |
| B | 2 | 4 | 4 | 4 | 4 | Papillary |
| C | 3 | 5 | 5 | 5 | 5 | Papillary |

**Table S2. Characteristics of nodules for whom all the 4 algorithms would have recommended to perform thyroid FNA**

| **Nodule** | **AACE/ACE/AME US** | **ACR-TIRADS** | **EU-TIRADS** | **K-TIRADS** | **SIAPeC-IAP classification** | **Pathology** |
| --- | --- | --- | --- | --- | --- | --- |
| D | 3 | 5 | 5 | 5 | 3B | Papillary |
| E | 3 | 5 | 5 | 5 | 4 | Papillary |
| F | 3 | 5 | 5 | 5 | 5 | Papillary |
| G | 3 | 5 | 5 | 5 | 5 | Papillary |
| H | 3 | 5 | 5 | 5 | 5 | Papillary |
| I | 3 | 5 | 5 | 5 | 4 | Papillary |

**Table S3. Estimation of the risk of thyroid malignancy according to nodule composition (solid versus mixed)**

| Pathology | Mixed composition | Solid composition | OR | p-value |
| --- | --- | --- | --- | --- |
| Non-malignant | 2 | 24 | +∞; 95%CI [0.28; +∞] | 0.1562 |
| Malignant | 0 | 39 |  |  |

**Table S4. Estimation of the risk of thyroid malignancy according to nodule shape (“taller than wide” versus round-ovoidal shape)**

| Pathology | Round-ovoidal shape | Taller than wide | OR | p-value |
| --- | --- | --- | --- | --- |
| Non-malignant | 21 | 5 | 1.1; 95%CI [0.27; 4.82] | 1 |
| Malignant | 31 | 8 |  |  |

**Table S5. Estimation of the risk of thyroid malignancy according to vascular signal (peri- and intranodular versus absent or peripheral)**

| Pathology | Absent or peripheral | Peri- and intranodular | OR | p-value |
| --- | --- | --- | --- | --- |
| Non-malignant | 23 | 3 | 1.96 95%IC [0.4; 12.7] | 0.503 |
| Malignant | 31 | 8 |  |  |
